# Neuroprotective Role of α-Lipoic Acid in Iron-Overload-Mediated Toxicity and Inflammation in In Vitro and In Vivo Models

**DOI:** 10.3390/antiox11081596

**Published:** 2022-08-18

**Authors:** Giuseppe Carota, Alfio Distefano, Mariarita Spampinato, Cesarina Giallongo, Giuseppe Broggi, Lucia Longhitano, Giuseppe A. Palumbo, Rosalba Parenti, Rosario Caltabiano, Sebastiano Giallongo, Michelino Di Rosa, Riccardo Polosa, Vincenzo Bramanti, Nunzio Vicario, Giovanni Li Volti, Daniele Tibullo

**Affiliations:** 1 Department of Biomedical and Biotechnological Sciences, University of Catania, 95123 Catania, Italy; giuseppe-carota@outlook.it (G.C.); distalfio@gmail.com (A.D.); mariaritaspampinato93@gmail.com (M.S.); lucialonghitano2891@gmail.com (L.L.); parenti@unict.it (R.P.); sebastiano.gll@gmail.com (S.G.); chitotriosidase@gmail.com (M.D.R.); d.tibullo@unict.it (D.T.); 2 Department of Scienze Mediche Chirurgiche e Tecnologie Avanzate “G.F. Ingrassia”, University of Catania, 95123 Catania, Italy; cesarina.giallongo@unict.it (C.G.); giuseppe.broggi@gmail.com (G.B.); palumbo.gam@gmail.com (G.A.P.); rosario.caltabiano@unict.it (R.C.); 3 Department of Clinical and Experimental Medicine, University of Catania, 95123 Catania, Italy; polosa@unict.it; 4 Division of Clinical Pathology, “Giovanni Paolo II” Hospital-A.S.P. Ragusa, 97100 Ragusa, Italy; vincenzo.bramanti@asp.rg.it

**Keywords:** iron, neurodegeneration, neuroinflammation, α-lipoic acid, antioxidant, microglia, zebrafish

## Abstract

Hemoglobin and iron overload is considered the major contributor to intracerebral hemorrhage (ICH)-induced brain injury. Accumulation of iron in the brain leads to microglia activation, inflammation and cell loss. Current available treatments for iron overload-mediated disorders are characterized by severe adverse effects, making such conditions an unmet clinical need. We assessed the potential of α-lipoic acid (ALA) as an iron chelator, antioxidant and anti-inflammatory agent in both in vitro and in vivo models of iron overload. ALA was found to revert iron-overload-induced toxicity in HMC3 microglia cell line, preventing cell apoptosis, reactive oxygen species generation and reducing glutathione depletion. Furthermore, ALA regulated gene expression of iron-related markers and inflammatory cytokines, such as IL-6, IL-1β and TNF. Iron toxicity also affects mitochondria fitness and biogenesis, impairments which were prevented by ALA pre-treatment in vitro. Immunocytochemistry assay showed that, although iron treatment caused inflammatory activation of microglia, ALA treatment resulted in increased ARG1 expression, suggesting it promoted an anti-inflammatory phenotype. We also assessed the effects of ALA in an in vivo zebrafish model of iron overload, showing that ALA treatment was able to reduce iron accumulation in the brain and reduced iron-mediated oxidative stress and inflammation. Our data support ALA as a novel approach for iron-overload-induced brain damage.

## 1. Introduction

Intracerebral hemorrhage (ICH) represents a leading cause of morbidity and mortality in the world, frequently associated with severe long-term disability in surviving patients [1]. ICH is the most common hemorrhagic stroke subtype, with an estimated incidence rate per year of 23.15 per 100,000 worldwide, with higher incidence in males [2]. Spontaneous and non-traumatic ICH is usually due to an underlying lesion, although most of the cases in the adult population are attributable to hypertension, which represents the most common systemic risk factor, reported in up to 70% of ICH patients [3]. In addition to symptomatic ICH, silent microbleeds in healthy adults could lead to asymptomatic conditions of ICH, with higher rate in older people; however, the long-term impact of such micro-hemorrhages is still not well defined [4]. ICH may be caused by several factors, including tumors, cerebral venous thrombosis, ruptured saccular aneurysms, inflammation and malformation [5,6], and is characterized by arteriole disruption in the brain, rapidly inducing primary brain injuries. In consequence, extravasated blood within the skull and mechanical compression increase intracranial pressure (ICP), leading to secondary brain injuries [1,7]. Despite a higher incidence associated with aging and the use of medications, such as anticoagulation and antiplatelet drugs, there are still only a limited number of suitable therapies to address ICH-derived primary and secondary damage [8,9]. The common approaches used to control primary brain injury include both surgical and minimally invasive measures to correct the coagulopathy and remove the presence of clots [10]. However, clinical trials have shown no significant amelioration after surgery, probably due to surgery-induced adverse effects [11,12]. The physiological response to edema and to the toxic effects mediated by clot components (i.e., hemoglobin/iron), lead to secondary brain injury [13,14]. It is well established that several clinical conditions are associated with iron overload, which are classified as primary or secondary forms, depending on whether the overload results from a primary defect in homeostasis of iron balance or it is secondary to other genetic or acquired disorders [15,16,17,18,19].

Iron release into the brain tissue typically occurs 24 h after hemorrhage; its deposition and accumulation into perihematomal tissue begins within a few days after an ICH, as a result of red blood cell lysis following ICH and the release of hemoglobin into the extracellular space [20,21,22,23]. The promotion of iron-mediated cell death is also fostered by microglia activation. Together with neutrophils, microglia release toxic substances, such as thrombin, ROS and metalloproteinases, leading to neuroinflammation and oxidative stress, which increase pro-inflammatory cytokines, such as tumor necrosis factor (TNF) and interleukin-6 (IL-6) [24,25]. In this context, it is evident that iron accumulation in the brain after ICH represents one of the main factors involved in inducing neurodegeneration, modulation of critical pathways for cell renewal and/or death, and long-term neurological deficits [26,27,28,29,30].

It has been shown that molecules serving as iron chelators and protease inhibitors, such as 1,2-dithiolane-3-pentanoic acid, known as α-lipoic acid (ALA), represent a potential therapeutic strategy for iron-overload-induced damage. ALA is an active organosulfur compound, naturally synthesized by plants and animals [31]. Both the oxidized (disulfide) and reduced (di-thiol: dihydro-lipoic acid, DHLA) forms of ALA show antioxidant properties. ALA exists as two different enantiomers: the biologically active (R)-isomer and the (S)-isomer. Commercial ALA is usually a racemic mixture of the R- and S-form [32]. ALA is both water and lipid soluble and is widely distributed in cellular membranes, the cytosol, and extracellular spaces and can cross the blood-brain barrier and cell monolayer in a pH-dependent manner, without causing any toxicity at therapeutic doses. The cellular transport of ALA occurs via several mechanisms, such as the medium-chain fatty acid transporter, an Na^+^-dependent vitamin transport system, and an H^+^-linked monocarboxylate transporter for intestinal uptake [33]. In the present study, we evaluated the efficacy of ALA, in both in vitro and in vivo models, on microglia exposed to ferric ammonium citrate (FAC).

## 2. Materials and Methods

### 2.1. Cell Culture and Treatments

HMC3 human microglial cells were purchased from the ATCC Company (Milan, Italy). Cells were cultured in minimum essential medium (MEM) supplemented with 10% fetal bovine serum (FBS), 100 U/mL penicillin, 100 U/mL streptomycin and 1% L-glutamine and were maintained in a humidified incubator at 37 °C with 95% air/5% CO_2_. At 80% confluency, cells were passaged using trypsin-EDTA solution (0.25% trypsin and 0.02% EDTA). The cells were pre-treated with ALA 100 μM for 3 h, while iron overload was achieved by exposing cells to FAC (Alfa Aesar-Thermofisher, Heysham, United Kingdom) 400 μM. Concentrations were selected according to previously published manuscripts by our group [34,35].

### 2.2. xCELLigence Real-Time Cell Analysis

An xCELLigence experiment was performed using a real-time cell analyzer dual plate platform according to the manufacturer’s instructions (Roche Applied Science, Mannheim, Germany; ACEA Biosciences, San Diego, CA, USA). Cells were seeded in E-16 xCELLigence plates at a final density of 5 × 10^3^ cells/mL per well. Plates were then incubated at 37 °C, 5% CO_2_ for 30 min to allow cell settling. After incubation, cells were treated with FAC 400 μM. Real-time changes in electrical impedance were measured and expressed as a “cell index”, defined as (Rn-Rb)/15, where Rn is the impedance of the well with cells and Rb is the background impedance. Background impedance was measured in an E-plate 16 with 100 μL medium (without cells) after a 30 min incubation period at room temperature. Cell proliferation was monitored every 20 min for 48 h.

### 2.3. Apoptosis Rate and Reactive Oxygene Species Analysis

Annexin V and propidium iodide (PI) staining were used to assess apoptosis. The evaluation was performed by flow cytometry. Samples (2 × 10^5^ cells) were washed twice and resuspended in 100 μL of PBS. Amounts of 1 μL of Annexin V-FITC solution and 5 μL of dissolved PI (Beckmam Coulter, Villepinte, France) were added to the cell suspension and mixed gently. Cells were incubated for 15 min in the dark. Finally, 400 μL of 1X binding buffer was added and cell preparation was analyzed by flow cytometry (MACSQuant Analyzer 10, Miltenyi Biotec).

Reactive oxygen species (ROS) were detected using 2′,7′-dichlorodihydrofluorescein acetate (H2-DCF; Sigma-Aldrich, St. Louis, MO, USA), and fluorescence intensity was measured according to the fluorescence detection conditions of FITC using a MACSQuant Analyzer (Miltenyi Biotech, North Rhine-Westphalia, Germany).

### 2.4. GSH Evaluation

The intracellular content of reduced glutathione (GSH) was measured using a spectrophotometric assay based on the reaction of thiol groups with 2,2-dithio-bis-nitrobenzoic acid (DTNB) at λ = 412 nm (εM = 13,600 M^−1^ × cm^−1^, where εM is a wavelength-dependent molar absorptivity coefficient). Measurements were performed in triplicate [36].

### 2.5. Mitochondrial Membrane Potential (DiOC2(3)) and Mass

A membrane potential probe, 3,3′-diethyloxacarbocyanine iodide (DiOC2(3)), (Thermo Fisher Scientific, Milan, Italy), was used to evaluate the mitochondrial membrane potential [37,38,39]. Cells were incubated with 10 μM DiOC2(3) for 30 min at 37 °C, washed twice, resuspended in PBS and analyzed by flow cytometry. To measure changes in the mitochondrial mass, cells were exposed to 200 nM MitoTracker Red CMXRos probe (Thermo Fisher Scientific, Milan, Italy) for 30 min at 37 °C, according to the manufacturer’s instructions. After two washes, labelled mitochondria were analyzed by flow cytometry.

### 2.6. Real-Time PCR for Gene Expression Analysis

RNA was extracted by Trizol^®^ reagent (Cat. no. 15596026, Invitrogen, Carlsbad, CA, USA). The first-strand cDNA was then synthesized with a high-capacity cDNA reverse transcription kit (Cat. no. 4368814, Applied Biosystems, Foster City, CA, USA). cDNA quality was checked, taking into consideration the housekeeping gene Ct values. Quantitative real-time PCR was performed in a step-one fast real-time PCR system (Applied Biosystems), using SYBR Green PCR MasterMix (Cat. no. 4309155, Life Technologies, Monza, Italy). PCR products were detected by the fluorescence of SYBR Green, a double-stranded DNA binding dye. Primers were designed using BLAST^®^ (Basic Local Alignment Search Tool, NCBI, NIH), considering the shortest amplicon proposed (primer sequences are shown in Table 1 and Table 2), and GAPDH was used as a housekeeping gene. Primers were purchased from Metabion International AG (Planneg, Germany). The relative mRNA expression level was calculated by the threshold cycle (Ct) value of each PCR product and normalized with GAPDH using a comparative 2^−ΔΔCt^ method.

### 2.7. ELISA

An ELISA test was used to measure the concentration of IL-6 in the medium conditioned by treatments. The assay was performed according to the manufacturer ‘s protocols (Cat. No BMS213INST, Invitrogen, Milan, Italy). Absorbance was measured at a wavelength of 450 nm and biomarker concentrations were calculated from a standard curve generated with purified proteins. The detection limit, as specified by the manufacturer, was 0.92 pg/mL. Each measurement was performed in triplicate.

### 2.8. Immunofluorescence

For immunofluorescence analysis, cells were plated on coverslips, exposed to treatments and processed as previously described [40,41,42,43,44]. After washing with PBS, cells were fixed in 4% paraformaldehyde (Sigma-Aldrich, Milan, Italy) for 20 min at room temperature. After fixation, cells were washed three times in PBS for 5 min and blocked in Odyssey blocking buffer for 1 h at room temperature. Subsequently, cells were incubated with mouse anti-arginase 1 (Cat. No. sc-271430, Santa Cruz Biotechnology, Heidelberg, Germany) and mouse anti-NOS2 (Cat. No. sc-7271, Santa Cruz Biotechnology), overnight at 4 °C. The following day, cells were washed three times in PBS for 5 min and incubated with the following secondary antibodies: anti-mouse TRITC-conjugated secondary antibody (Cat. No. A11003, ThermoFisher Scientific, Milan, Italy), and anti-mouse FITC-conjugated secondary antibody (Cat. No. F2012, Sigma-Aldrich, St. Louis, MO, USA) for 1 h at room temperature. Antibodies were diluted in Odyssey blocking buffer. Slides were mounted with mounting medium containing 4,6-diamidino-2-phenylindole (DAPI, Cat. No. D1306, Invitrogen, 1:1000) to highlight nuclei. Fluorescent images were obtained using a Zeiss Axio Imager Z1 microscope with an Apotome 2 system (Zeiss, Milan, Italy).

### 2.9. Iron-Overload Zebrafish Model

Adult wildtype AB zebrafish (*n* = 20) were used for this study. Fish were tested to be free from *Pseudoloma neurophilia*, *Pseudocapillaria tomentosa*, *Mycobacterium* spp., and *Edwardsiella ictalurias*, determined by twice-yearly sentinel monitoring. The fish were housed at a density of 5 fish per tank in mixed-sex groups in 2.5 L tanks on a recirculating system in 28 °C water in a room with a 14:10 h light/dark cycle. System water was carbon-filtered municipal tap water, filtered through a 20 μm pleated particulate filter, and exposed to 40 W UV light. The standard feeding protocol was three meals daily of Tetra-Min (Tetra) at a Center for Advanced Preclinical in vivo Research (CAPIR, University of Catania) facility. All zebrafish experiments were performed with the approval of the Animal Studies Committee of Ministero della Salute Italy (Approval code: 813/2017-PR, 23 October 2017). Each subject was randomly assigned to the following experimental group: controls, FAC 400 μM, ALA 100 μM, FAC 400 μM + ALA 100 μM. The chemical agents tested were introduced into a static 2 L tank filled with system water obtained from the main recirculating system. A control group of untreated fish housed under the same conditions as the experimental groups was tested in parallel for 120 h. Experiments were performed after pre-treating animals with FAC 400 μM for 60 h and then exposing them to ALA 100 μM up to the end of the experiment.

### 2.10. Immunoistochemistry

Zebrafish brains were isolated, fixed in 10% buffered-formaldehyde, dehydrated in increasing concentrations of ethanol in water and paraffin-embedded, preserving their anatomical orientation [45,46,47,48,49]. Three-to-four-μm-thick sections were obtained using a microtome and mounted on silane-coated slides. GFAP staining was performed as previously reported [50,51,52]. Briefly, rehydrated sections were blocked with a blocking solution (3% H_2_O_2_ in PBS) for 15 min at room temperature in a humidity chamber and incubated for 40 min at room temperature in a humidity chamber with a mouse anti-GFAP antibody (1:100, Cat. No ab154474). After washes, sections were incubated with biotinylated panspecific secondary antibody (horse anti-mouse/rabbit/goat IgG Antibody (H + L), Cat.No BA-1300-2.2), diluted in PBS and 1% bovine serum albumin (BSA, Sigma-Aldrich, Cat.No. A2058) for 30 min at room temperature in a humidity chamber. Then VECTASTAIN^®^ Elite ABC-HRP Reagent, Peroxidase, R.T.U. (Vector Laboratories, Milan, Italy, Cat.No PK-7100) was added and sections were incubated for 30 min at room temperature in a humidity chamber. A solution of 1% DAB and 0.3% H_2_O_2_ in PBS was added until brown coloration was observed. Then, slides were washed in tap-water for 5 min. Nuclei were counterstained with Mayer’s hematoxylin (Bio-Optica, Milan, Italy), dehydrated with increasing concentrations of ethanol (50%, 70%, 95%, 100%) and xylene, and cover-slipped with Entellan (Cat.No. 1.079.600.500, Merck Millipore, Milan, Italy). Digital images were acquired using a Nexcope NIB600 biological microscope.

For Perl’s Prussian Blue staining (Bio-Optica, Milan, Italy), slides were dewaxed in xylene and re-hydrated using decreasing concentrations of ethanol in water (100%, 75%, 50% and 0%). Perl’s Prussian Blue staining was performed according to the manufacturer’s instructions. Briefly, tissue sections were washed in water and stained with Perl’s staining for 20 min. Sections were then rinsed in water, dehydrated in increasing concentrations of ethanol in water (0%, 50%, 75%, 100%), cleared in xylene, and finally mounted for microscopic analysis.

### 2.11. Statistical Analysis

Data are shown as mean ± standard deviation (SD). For statistical analysis, Prism 8.0.2. software (GraphPad Software, San Diego, CA, USA) was used. Significant differences between groups were assessed using a one-way ANOVA test. A value of *p* < 0.05 was considered statistically significant and symbols used to indicate statistical differences are described in figure legends.

## 3. Results

### 3.1. Iron Overload Affects Cell Survival and Iron-Metabolism-Related Gene Expression

**Figure 1:**
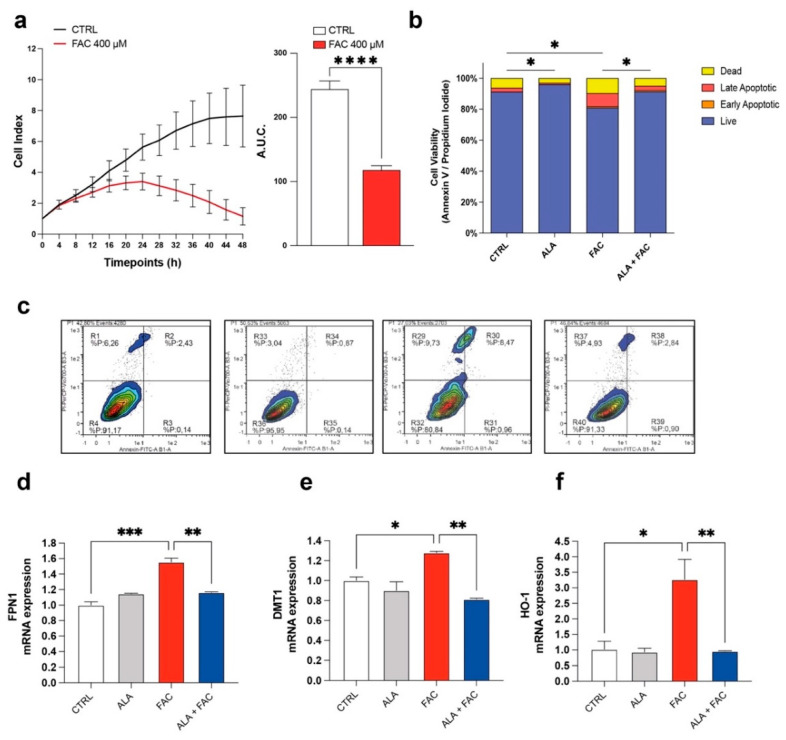
Iron overload affects cell survival and iron-metabolism-related gene expression. (**a**) Time course cell index and area under the curve (A.U.C.) of control HMC3 cells and HMC3 cells treated with 400 μM of FAC. (**b**,**c**) Quantification (**b**) and representative dot plots (**c**) of cell viability of control, ALA-, FAC- and ALA + FAC-treated HMC3 cells; the proportion of live, early and late apoptotic cells and dead cells are reported. (**d**,**f**) Quantification of mRNA expression levels of FPN1 (**d**), DMT1 (**e**) and HO-1 (**f**) in control, ALA-, FAC- and ALA + FAC-treated HMC3 cells. * *p*-value < 0.05; ** *p*-value < 0.01; *** *p*-value < 0.001 and **** *p*-value < 0.0001.

To confirm the toxicity induced by iron overload on cell proliferation, dynamic changes in microglia cell proliferation were monitored for 48 h using the xCELLigence system upon exposure to FAC 400 μM. We observed that FAC affected the proliferation rate of the HMC3 microglia cell line after 8 h. After 24 h, the proliferation rate of treated cells was decreased compared to untreated control cells (Figure 1a). Moreover, the apoptosis rate, measured by flow cytometry using Annexin V and propidium iodide (PI), showed an increase in apoptotic cell number in HMC3 cells treated with FAC (Figure 1b,c). Interestingly, 3 h pre-treatment with ALA 100 μM [34] was able to prevent the cell death induced by FAC treatment after 24 h, and ALA in cotreatment with FAC showed a near normal Annexin/PI profile (Figure 1b,c). To observe specific changes in iron metabolism gene expression, HMC3 cells were pre-treated with ALA and then exposed to FAC for 6 h. Analysis of mRNA levels revealed that iron-metabolism-related mediators, such as, heme oxygenase 1 (HO-1), ferroportin 1 (FPN1) and divalent metal transporter 1 (DMT1, iron uptake), were significantly upregulated in the presence of FAC and, importantly, ALA was able to prevent this phenomenon (Figure 1d,e).

### 3.2. Iron Overload Affects Mitochondrial Functionality Inducing Oxidative Stress

**Figure 2:**
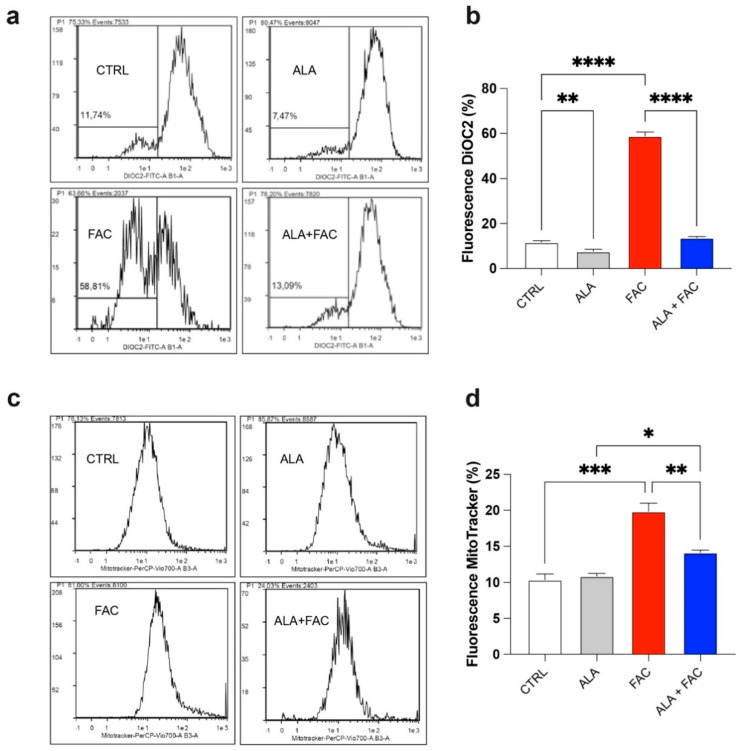
Iron overload increases mitochondrial mass in HMC3 cell line. (**a**,**b**) Representative plots (**a**) and quantification (**b**) of DiOC2 fluorescence intensity via cytofluorimetric analysis in control, ALA-, FAC- and ALA + FAC-treated HMC3 cells; (**c**,**d**) Representative plots (**c**) and quantification (**d**) of mitotracker fluorescence intensity via cytofluorimetric analysis in control, ALA-, FAC- and ALA + FAC-treated HMC3 cells. * *p*-value < 0.05; ** *p*-value < 0.01; *** *p*-value < 0.001; **** *p*-value < 0.0001.

It is well known that iron can alter mitochondrial functionality by increasing the depolarization of the mitochondrial membrane and inducing ROS production. This generates a cellular response aimed at maintaining mitochondrial homeostasis by increasing mitochondrial biogenesis. We found that FAC-induced iron overload of the microglial cell line induced a high level of depolarization compared to untreated healthy cells (Figure 2a,b), whereas the combined presence with ALA restored the membrane potential (Figure 2a,b). As expected, we also observed an increase in the mitochondrial mass after FAC treatment (Figure 2c,d); this effect was restored by pre-treatment with ALA for 24 h (Figure 2c,d).

**Figure 3:**
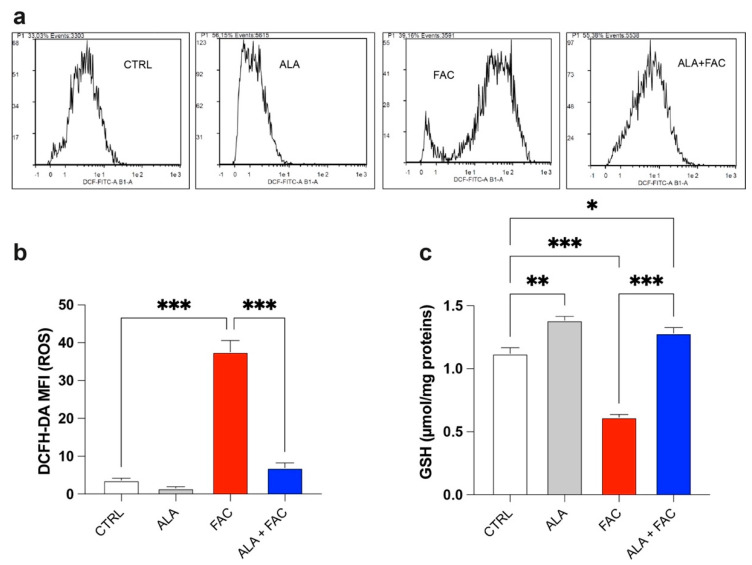
Iron overload affects mitochondrial functionality inducing oxidative stress. (**a**,**b**) Representative plots (**a**) and quantification (**b**) of DCFH-DA fluorescence intensity via cytofluorimetric analysis in control, ALA-, FAC- and ALA + FAC-treated HMC3 cells; (**c**) Quantification of intracellular content of reduced GSH analyzed by spectrophotometric assay in control, ALA-, FAC- and ALA + FAC-treated HMC3 cells. * *p*-value < 0.05; ** *p*-value < 0.01; *** *p*-value < 0.001.

The quantitative measurement of cells undergoing oxidative stress and ROS production was evaluated by flow cytometry. Treatment with FAC led to a strong increase in ROS production in HMC3 cells compared to the control group. On the other hand, iron overload in the presence of ALA was not able to induce the same amount of ROS content (Figure 3a,b). This effect was confirmed by the higher glutathione (GSH) levels observed in the presence of iron overload. Furthermore, in this case, the ALA treatment was able to counteract the oxidative stress induced by FAC with decrease in GSH levels (Figure 3c).

### 3.3. Iron Overload and Inflammation

**Figure 4:**
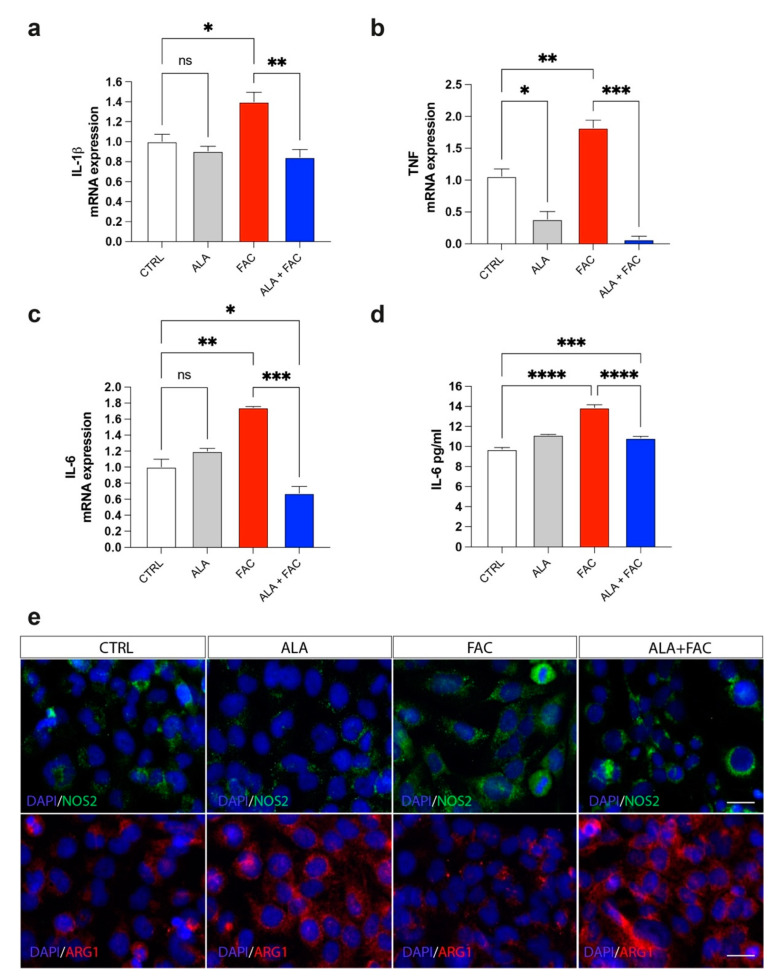
Iron overload induces microglia inflammatory phenotype reverted by ALA. (**a**–**c**) Quantification of mRNA expression levels of IL-1β (**a**), TNF (**b**) and IL-6 (**c**) in control, ALA-, FAC- and ALA + FAC-treated HMC3 cells. (**d**) ELISA of IL-6 levels in the supernatant of control, ALA-, FAC- and ALA + FAC-treated HMC3 cells. (**e**) Representative pictures of immunofluorescence analysis of NOS2 and ARG1 in control, ALA-, FAC- and ALA + FAC-treated HMC3 cells. * *p*-value < 0.05; ** *p*-value < 0.01; *** *p*-value < 0.001; **** *p*-value < 0.0001.

Iron overload should generate a significant inflammatory state in all tissues in which it is possible to detect this pathological state. The exposure to iron caused an increase in markers closely related to inflammatory genes, such as prostaglandin-endoperoxide synthase (COX-2) and interleukins (IL-6, IL-1β) (Figure 4a–c). Due to the pre-treatment with ALA, the expression of these inflammatory genes was reduced to the control level (Figure 4a–c). These data were also confirmed by ELISA assay for the evaluation of IL-6 concentration in the medium of HMC3 cells treated with FAC alone or in combination with ALA. We observed an increase in IL-6 levels after exposure with FAC, which was reverted by ALA co-treatment (Figure 4d). Interestingly, the M1-like or M2-like phenotype in microglia was characterized by over expression of iNOS (NOS2) or arginase 1 (ARG1), respectively. We observed, by immunofluorescence assay, that FAC-exposed cells showed a pro-inflammatory state in microglia cells, as shown by increase in iNOS protein expression, while ARG1 levels were mostly unchanged (Figure 4e). Of note, ALA alone was able to produce strong overexpression of ARG1 (Figure 4e). Finally, pre-treatment with ALA prevented inflammatory effects induced by FAC exposure; in addition, ALA was able to mediate an anti-inflammatory response, supporting an M2-like anti-inflammatory phenotype in microglia cells (Figure 4e).

### 3.4. Iron Overload In Vivo Zebrafish Model

**Figure 5:**
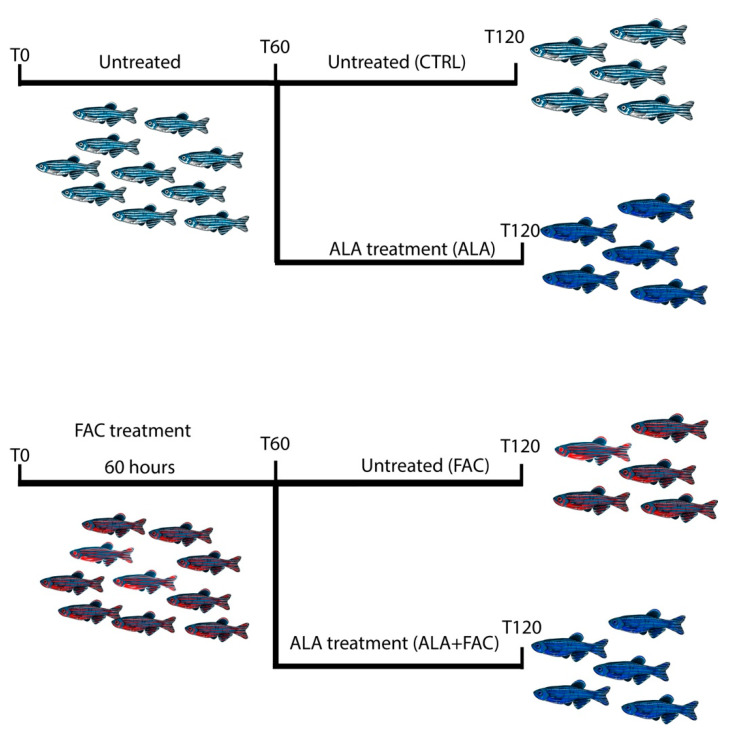
Schematic representation of the experimental design of zebrafish model of iron overload and ALA treatment.

**Figure 6:**
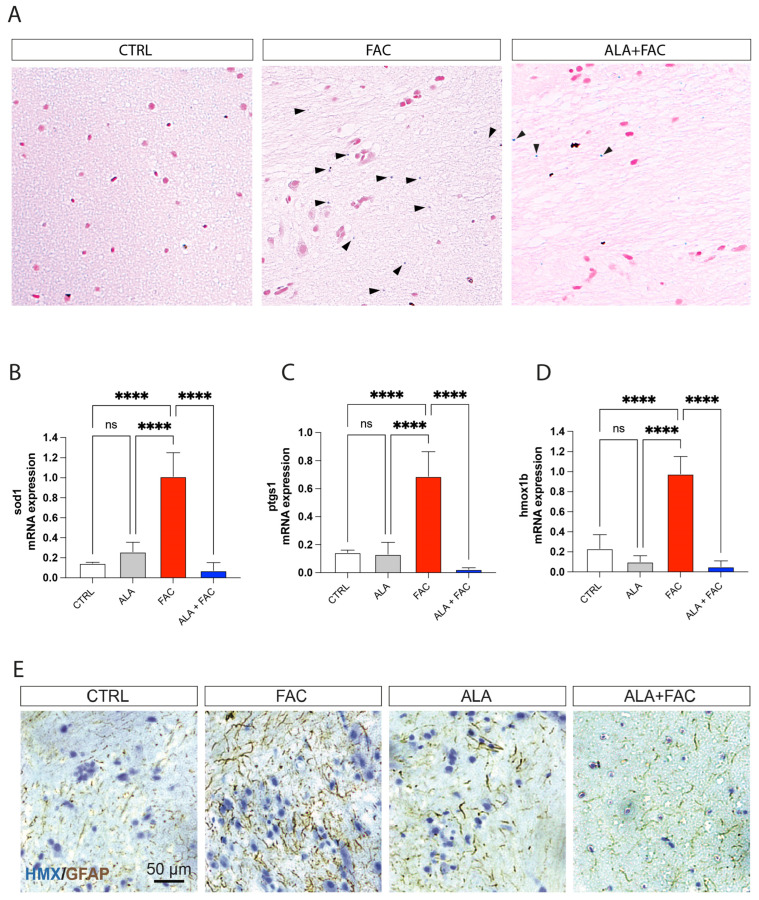
Iron exposition induces iron accumulation and gliosis in zebrafish brain. (**A**) Representative pictures of the brain stained with Perl’s Prussian Blue staining in control, FAC- and ALA + FAC-exposed zebrafish brain. (**B**–**D**) Quantification of mRNA expression levels of sod1 (**A**), ptgs1 (**B**) and hmox1b (**C**) in control, ALA-, FAC- and ALA + FAC-exposed zebrafish. (**E**) Representative pictures of the brain stained with hematoxylin and GFAP of control, FAC-, ALA- and ALA + FAC-exposed zebrafish. **** *p*-value < 0.0001.

To confirm the effect of ALA in an iron overload model, we treated adult zebrafish with 400 μM of FAC for 60 h and then with ALA 100 μM (Figure 5). As shown by whole brain immunostaining of iron using Perl’s staining, FAC treatment resulted in significant brain iron deposition compared to control animals. The following administration of ALA, after 60 h of FAC exposure, resulted in a reduction in iron content (Figure 6A). We also evaluated, in the whole brain, the expression of inflammatory and oxidative-stress-related genes. The FAC-exposed group showed a significant increase in hmox1b, sod1 and COX-1 (ptgs1) expression indicating that iron accumulation was able to produce an inflammatory and then oxidative state in the zebrafish brain (Figure 6B–D). Interestingly, as for the in vitro model, ALA was able to counteract FAC-induced effects (Figure 6B–D). These results were coupled with reduced expression of GFAP positive cells in the brain of FAC-exposed ALA-treated zebrafish compared to FAC alone (Figure 6E).

## 4. Discussion

Iron homeostasis is an essential factor in maintaining physiological functions. High levels of iron are responsible for ROS production that, by overwhelming the physiological cellular antioxidant system, leads to cellular damage and organ failure. The main target of iron toxicity is the liver, due to its predominant role in iron metabolism. However, iron accumulation also leads to toxicity to a number of other tissues, such as bone marrow, the intestine, and the central nervous system.

In the central nervous system, iron dysregulation can lead to significant neuronal damage and neurodegenerative conditions, such as Alzheimer’s disease, Parkinson’s disease and neuropathies [53,54,55,56]. Although metal homeostasis is recognized as an important process in these kinds of brain diseases, the role of iron accumulation following ICH or traumatic brain injury has not been fully elucidated. Both the latter conditions are broadly defined as bleeding within the cranium, leading to different brain injuries categorized according to severity and prognosis. Few Food and Drug Administration (FDA)-approaved chelating agents are currently clinically available.

On the one hand, deferoxamine is the most used iron chelator, with the ability to prevent neural damage and to reduce perihematomal edema occurrence, with a number of studies suggesting its use for its neuroprotective role in ICH conditions [57,58,59,60]. On the other hand, limited evidence is available on the effect of deferoxamine on neurologic outcomes after ICH and there are concerns with its safety profile, particularly due to neurotoxicity associated with repeated exposure [61,62,63]. Beyond ICH, iron overload affecting brain functionalities may occur under various and collateral pathological conditions, including genetic conditions, such as hereditary haemochromatosis, or acquired conditions that require lifelong regular blood transfusions, such as thalassemia [64,65,66]. More than 10% of patients affected by these conditions and treated with deferoxamine experience severe adverse effects, such as retinal and auditory neurotoxicity, neutropenia and agranulocytosis, diarrhea, headache, nausea, abdominal pain, increased serum creatinine, increased liver enzymes, rash, fatigue, and arthralgia [67]. To address the clinical need, we assessed ALA properties in both in vitro and in vivo models of iron overload.

It is well-known that iron accumulation can affect microglia proliferation and viability, leading to an overall degeneration of functionalities [68,69,70]. Our in vitro results confirmed a strong reduction in cell proliferation after treatment with FAC which was also able to influence the apoptotic rate of microglia cells. FAC led to intracellular iron accumulation, shown by increased levels of FPN1 and DMT1, resulting in significant impairment of mitochondrial membrane potential, thereby causing an increased rate of ROS generation and cell damage. Our results showed that pre-treatment with ALA prevented FAC toxicity by protecting cells, both as an iron chelator and as an antioxidant molecule. We observed that ALA treatment reduced the expression of the iron transporters FPN1 and DMT1, likely inducing reduced iron intake. This effect was coupled with reduced oxidative stress, both enzymatically and through a direct free-radical-scavenging effect [71,72]. ALA, as a dithiol compound, is able to bind lysine residues of mitochondrial α-keto acid dehydrogenases, which reduce ALA to dihydrolipoic acid (DHLA), known for its strong antioxidant properties [73]. The presence of ALA was able to maintain low ROS levels in microglia, to reduce iron content and to restore mitochondrial membrane potential and integrity. Our results were further confirmed by changes in both intracellular GSH content and in HO-1 expression.

The GSH system is the main cellular defense mechanism regulating intracellular redox state and its synthesis is promptly activated by several oxidative triggers [74]. Under physiological conditions, the reduced GSH is the major form available with a concentration ranging from 10- to 100-fold higher than the oxidized form, thus ensuring oxidative cell balance [75]. Our results showed that treatment with FAC decreased the amount of reduced GSH available, while ALA pre-treatment enhanced the antioxidant defenses for the cells by restoring the GSH pool. In parallel, the results showed that HO-1, an NRF2 regulated protein involved in redox balance, was strongly upregulated following FAC treatment. HO-1 plays a pivotal role in regulating redox homeostasis via anti-inflammatory, antioxidant and anti-apoptotic properties [76,77,78]. It has been reported that the activation of HO-1 is a ubiquitous cellular response to oxidative stress and acts to stimulate ferritin synthesis, which ultimately contributes to iron detoxification [79,80]. Consistent with this evidence, we observed that iron overload in microglia triggered the activation of cellular defenses through upregulation of HO-1, while co-treatment with ALA was able to prevent activation of this pathway. This effect may be linked to the restored GSH content, counteracting reactive oxygen species. As shown, cellular stress caused by FAC exposure affects mitochondrial functions and homeostasis through the impairment of the membrane potential. According to Hoeft et al., macrophages exposed to iron overload enhanced mitochondrial mass, increasing the proportion of non-functional mitochondria over total mitochondrial content [81]. These authors suggested that mitochondrial mass can be increased by promoting mitochondrial biogenesis and/or by decreasing mitophagy, and reported that treating cells with a combination of lipopolysaccharide and iron (as further sources of cellular stress) increased the mRNA levels of genes involved in mitochondrial biogenesis [81]. Furthermore, a large body of evidence has shown that, although the total mitochondrial mass is increased, damaged or non-functional mitochondria induce increased ROS levels [82,83]. Our results largely confirmed that iron overload in microglia led to an increase in total mitochondrial mass, although new mitochondria were likely to be non-functional, as shown by abnormal ROS production and by mitochondrial membrane depolarization. Moreover, mitochondrial biogenesis was confirmed by the significantly high levels of related genes, such as ATP5B, CYTB and TFAM. It is worth noting that mitochondrial biogenesis is regulated by nuclear transcription factors, such as NRF1 and NRF2, and the coactivator peroxisome proliferator-activated receptor γ coactivator 1 (PGC1α), which upregulates TFAM, enabling replication of mitochondrial DNA [84,85,86]. As such, we can speculate that oxidative stress caused by iron overload is able to activate HO-1, as well as to affect mitochondrial biogenesis, probably through a shared signaling pathway involving nuclear translocation of NRF2 and binding to antioxidant response elements in the nuclear DNA. ALA treatment reverted the effect of FAC on mitochondria due to its action as a chelator, but also by exerting antioxidant effects via NRF2/HO-1 pathway activation [87].

Iron overload and inflammation are linked by bidirectional crosstalk. Iron modifies the inflammatory phenotype of microglia and infiltrating macrophages, and, in turn, these cells secrete diffusible mediators, reshaping neuronal iron homeostasis and regulating iron entry into the brain. Our results showed that iron-exposed microglial cells rapidly switched toward a pro-inflammatory phenotype, as highlighted by NOS2 over-expression. This data was confirmed by pro-inflammatory cytokines and inflammation-related marker increase, including IL-6, TNF, IL-1β and COX-2. Interestingly, pre-treatment with ALA prevented the inflammatory state, not only by reducing pro-inflammatory cytokine expression, but also by counteracting NOS2 expression and enhancing ARG1 levels, a marker of the anti-inflammatory microglia phenotype. Our results were also in accordance with evidence published by Su Min Kim and coworkers, showing that ALA treatment reduced M1-like phenotype markers in LPS-challenged BV2 cells, whereas the M2-like anti-inflammatory phenotype was significantly enhanced [88]. Our in vitro evidence demonstrated that pre-treatment with ALA strengthens microglia cells by protecting them from iron-overload-induced toxicity.

However, since ICH and linked iron accumulation can be considered unpredictable events, the pre-administration of ALA used in in vitro models can only provide conceptual answers, but cannot fully overlap with clinical conditions, in which a therapeutic regimen is given after several hours upon ICH occurrence. For this reason, to test ALA efficacy in a translational model, we developed an in vivo iron overload model based on the acute exposure of zebrafish to FAC, administrating ALA after iron accumulation.

The zebrafish is an important vertebrate model used in research due to its combination of excellent embryological characteristics and susceptibility to genetic manipulation. It has been shown that the uptake of iron in zebrafish occurs via absorption from water across the gill membrane and intestinal mucosa [89]. Due to this mechanism, there is the possibility for iron to be lost through the gills by diffusion following a concentration gradient, for instance, when zebrafish are transferred to water with a lower concentration of iron [90]. To ensure that every change in iron content or in gene expression was due to the efficacy of ALA as an iron chelator and antioxidant, water in FAC-exposed or ALA + FAC groups was replaced with untreated water. Our data demonstrated that ALA was able to strongly reduce iron content accumulated in the brain compared to FAC alone. Furthermore, because of its direct and indirect antioxidant properties, and its efficacy in inflammatory conditions, ALA resulted in a significant reduction in hmox1b, sod1, ptgs1 gene expression in the brain when compared to FAC treatment. Therefore, our results are consistent with those previously obtained by our group, in which the efficacy of ALA on iron storage reduction in the zebrafish liver and intestine was demonstrated [34]. With our new data, we demonstrated that ALA was able to exert its activity as an iron chelator in brain tissue, without showing any loss of effectiveness on iron overload.

## 5. Conclusions

In conclusion, we suggest that ALA can exert antioxidant, anti-inflammatory and iron-chelating properties both in vitro and in vivo. Since there are still many issues regarding the availability of reliable therapies to respond to ICH following iron overload, we suggest that ALA could be considered, in the near future, as a new therapeutic opportunity with great potential, particularly considering its observed low adverse effects.

## Figures and Tables

**Table 1:** Primer sequences used to assess gene expression in human cells.

Gene (Human)	Forward Primer (5′ → 3′)	Reverse Primer (5′ → 3′)
HO-1	GTTGGGGTGGTTTTTGAGCC	TTAGACCAAGGCCACAGTGC
DMT1	CGGAATAGGAAGTGCCATCCA	GGGAGCAAGGAAAAAGAACTACA
FPN1	CTCCCAAACCGCTTCCATAAG	TCTTCTGCGGCTGCTATCG
IL-6	CCACCGGGAACGAAAGAGAA	GAGAAGGCAACTGGACCGAA
IL-1β	AGCTCGCCAGTGAAATGATG	GTCGGAGATTCGTAGCTGGA
TNF	GCAACAAGACCACCACTTCG	GATCAAAGCTGTAGGCCCCA
GAPDH	TTCTTTTGCGTCGCCAGCC	CTTCCCGTTCTCAGCCTTGAC

**Table 2:** Primer sequences used to assess gene expression in zebrafish brain lysate.

Gene (Zebrafish)	Forward Primer (5′ → 3′)	Reverse Primer (5′ → 3′)
hmox1b	CTCTCCAGCCCTTCAGTTCG	AAGCGTAAACTCCCATGCCA
sod1	GTGACAACACAAACGGCTGC	GGCATCAGCGGTCACATTAC
ptgs1	ACTTTACCACTGGCACCCAC	ACGATGACCCTCTCAGCAAC
gapdh	CATCTTTGACGCTGGTGCTG	TGGGAGAATGGTCGCGTATC

## Data Availability

The data presented in this study are available in the article.
