# Correction: Carota et al. Neuroprotective Role of α-Lipoic Acid in Iron-Overload-Mediated Toxicity and Inflammation in In Vitro and In Vivo Models. *Antioxidants* 2022, *11*, 1596

**DOI:** 10.3390/antiox12122099

**Published:** 2023-12-12

**Authors:** Giuseppe Carota, Alfio Distefano, Mariarita Spampinato, Cesarina Giallongo, Giuseppe Broggi, Lucia Longhitano, Giuseppe A. Palumbo, Rosalba Parenti, Rosario Caltabiano, Sebastiano Giallongo, Michelino Di Rosa, Riccardo Polosa, Vincenzo Bramanti, Nunzio Vicario, Giovanni Li Volti, Daniele Tibullo

**Affiliations:** 1 Department of Biomedical and Biotechnological Sciences, University of Catania, 95123 Catania, Italy; giuseppe-carota@outlook.it (G.C.); distalfio@gmail.com (A.D.); mariaritaspampinato93@gmail.com (M.S.); lucialonghitano2891@gmail.com (L.L.); parenti@unict.it (R.P.); sebastiano.gll@gmail.com (S.G.); chitotriosidase@gmail.com (M.D.R.); d.tibullo@unict.it (D.T.); 2 Department of Scienze Mediche Chirurgiche e Tecnologie Avanzate “G.F. Ingrassia”, University of Catania, 95123 Catania, Italy; cesarina.giallongo@unict.it (C.G.); giuseppe.broggi@gmail.com (G.B.); palumbo.gam@gmail.com (G.A.P.); rosario.caltabiano@unict.it (R.C.); 3 Department of Clinical and Experimental Medicine, University of Catania, 95123 Catania, Italy; polosa@unict.it; 4 Division of Clinical Pathology, “Giovanni Paolo II” Hospital-A.S.P. Ragusa, 97100 Ragusa, Italy; vincenzo.bramanti@asp.rg.it

In the original publication [1], there was a mistake in Figure 6 as published. In Figure 6a, one of the panels was accidentally duplicated. For this reason, we want to upload a new version where we amend this error. The corrected Figure 6 appears below. The authors state that the scientific conclusions are unaffected. This correction was approved by the Academic Editor. The original publication has also been updated.

## Figures and Tables

**Figure 6:**
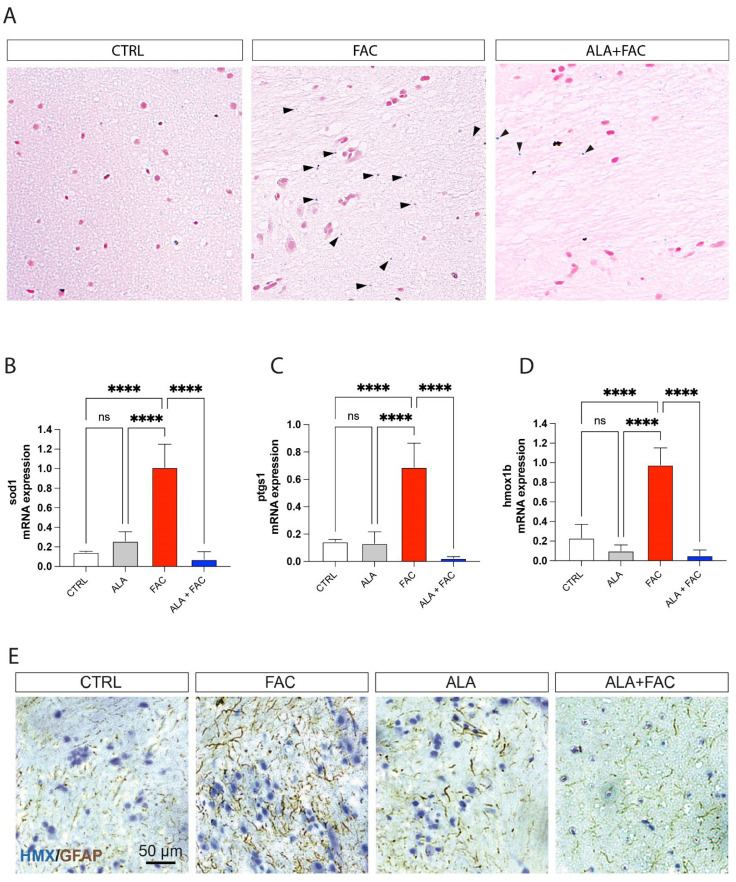
Iron exposition induces iron accumulation and gliosis in zebrafish brain. (**A**) Representative pictures of the brain stained with Perl’s Prussian Blue staining in control, FAC- and ALA + FAC-exposed zebrafish brain. (**B**–**D**) Quantification of mRNA expression levels of sod1 (**A**), ptgs1 (**B**) and hmox1b (**C**) in control, ALA-, FAC- and ALA + FAC-exposed zebrafish. (**E**) Representative pictures of the brain stained with hematoxylin and GFAP of control, FAC-, ALA- and ALA + FAC-exposed zebrafish. **** *p*-value < 0.0001.
